# *IL1R1* gene polymorphisms are associated with knee osteoarthritis risk in the Chinese Han population

**DOI:** 10.18632/oncotarget.13935

**Published:** 2016-12-14

**Authors:** Yuyan Na, Rui Bai, Zhenqun Zhao, Yishan Wei, Daihe Li, Yong Wang, Chao Sun, Liang Sun, Bolun Zhang, Tianbo Jin, Wanlin Liu

**Affiliations:** ^1^ Department of Pediatric Orthopedics, The Second Affiliated Hospital of Inner Mongolia Medical University, Hohhot, Inner Mongolia Autonomous Region, China; ^2^ Graduate School of Inner Mongolia Medical University, Hohhot, Inner Mongolia Autonomous Region, China; ^3^ Xi’an Tiangen Precision Medical Institute, Xi’an, Shaanxi, China; ^4^ National Engineering Research Center for Miniaturized Detection Systems, School of Life Sciences, Northwest University, Xi’an, Shaanxi, China

**Keywords:** knee OA, *IL1R1*, genetic polymorphism, case-control study

## Abstract

*IL1R1*, encoding interleukin 1 receptor type 1, is located in the *IL-1* gene cluster and is involved in the pathogenesis of hand, hip, and knee osteoarthritis (OA) in different ethnicities. However, the link between *IL1R1* polymorphisms and OA risk in the Chinese Han population is unknown. We studied the association between five *IL1R1* polymorphisms (rs10490571, rs12712127, rs956730, rs3917225, and rs3917318) and OA risk by analyzing the genotypes of 298 knee OA patients and 297 controls using Sequenom MassARRAY technology. Logistic regression analysis after adjusting for gender and age revealed significant differences in the allele frequencies of *IL1R1* rs956730 and *IL1R1* rs3917225 between patients and controls. In addition, *IL1R1* rs3917225 was associated with increased risk of knee OA with or without adjustment by age and gender in the dominant model (adjusted OR= 1.47, 95%CI: 1.04-2.07, *P* = 0.030), the recessive model (adjusted OR= 1.75, 95%CI: 1.08-2.85, *P*= 0.023), and the additive model (adjusted OR= 1.40, 95%CI: 1.09-1.79, *P* = 0.007). This study is the first to report that *IL1R1* polymorphisms are associated with knee OA susceptibility in the Northwestern Chinese Han population.

## INTRODUCTION

Osteoarthritis (OA) is an age-related degenerative disorder that predominantly occurs in the middle aged and older people. Recent evidence demonstrates that OA is an inflammatory disease that affects large weight-bearing joints, such as hips and knees [1]. OA pathology includes articular cartilage destruction, attrition of subchondral bone, and abnormal remodeling which induces joint pain, stiffness, swelling and eventually results in disability [2]. While factors like old age, gender (prominent in females), obesity, nutrition, severe injury, and repetitive joint loading increase predisposition to OA, genetic factors are a major determinant of this illness [3–6].

Interleukin 1 (IL-1) is a pleiotropic cytokine involved in the inflammatory process of OA. The secretion of IL-1β, the active form of IL-1 during inflammation, is increased in the OA-affected cartilage and synovial cells [7]. Moos and others found a 10-fold up-regulation of IL-1β protein and mRNA in human OA-affected cartilage compared to normal articular cartilage [8]. *IL1R1*, that encodes cytokine receptor for IL1 is located on 2q12.1. It affects NF-κB signaling by combining with IL-1 on the cell surface and upregulates inflammation [9]. Mukundan and others reported IL-1R1 mRNA expression in human OA-affected cartilage [7]. Further, OA progression in osteoarthritic rabbit knee joints was shown to be prevented by blocking IL-1R1 signal transduction [10]. A genome wide scan by Leppavuori and others identified a candidate region on chromosome 2q harboring the *IL-1* gene cluster for distal interphalangeal joint OA in a Finnish population [11]. Meulenbelt and colleagues demonstrated the role of *IL-1* gene cluster variants in the pathogenesis of hip OA [12]. Smith and others found that linkage disequilibrium in the *IL1R1-IL1A-IL1B-IL1RN* gene cluster was associated with knee OA in a British study [13]. Nakki and colleagues performed a single SNP association analysis and reported four SNPs (rs1465325, rs956730, rs3917225, and rs2287047) in the *IL1R1* that were associated with severe hand OA in a Finnish population [14]. Since these studies showed the genetic link between *IL1R1* and OA in defined patient populations, the aim of our study was to evaluate the role of *IL1R1* polymorphisms with knee OA risk in the Northwestern Chinese Han population.

## RESULTS

### Population characteristics

The distributions of age and gender of the 298 knee OA patients and 297 controls are listed in Table 1. Since there were differences in age between the two groups (*P*< 0.001), the variable for age and gender was adjusted in multivariate unconditional logistic regression analysis to eliminate residual confounding effects.

**Table 1:** Distributions of age and gender in knee OA patients and controls

Variable	Patients	Controls	*P* value
	(*n*=298)	(*n*=297)	
Gender			> 0.05
Male	99 (33.2%)	99 (33.3%)	
Female	199 (66.8%)	198 (66.7%)	
Age(years)	60.60	56.35	< 0.001
Std. Deviation	4.80	9.22	

*P* values were calculated from Pearson Chi-square test/ Welch's *t* test.

### Allele and genotype frequency distribution between knee OA patients and controls

The allele and genotype frequencies of the *IL1R1* polymorphisms are presented in Tables 2 and 3. Our analysis showed that *IL1R1* rs12712127 deviated from the Hardy-Weinberg equilibrium (HWE) (*P*< 0.0001) and hence was removed (Table 2). The genotype distributions of the remaining SNPs in controls did not show any difference from those expected under HWE (*P*> 0.05). The frequency of the “A” allele of *IL1R1* rs956730 was different for patients in comparison to controls (21.3% versus 26.8%) and showed decreased risk (OR= 0.74, 95%CI: 0.57-0.97, *P*= 0.028). On the other hand, the frequency of the “G” allele of *IL1R1* rs3917225 was also different for patients in comparison to controls (41.3% versus 33.7%). and showed increased risk (OR= 1.38, 95%CI: 1.09-1.75, *P*= 0.007) of knee OA (Table 3). In accordance with the allele data, the frequency of homozygous variants “GG” genotype of *IL1R1* rs3917225 differed significantly between the two groups (18.1% versus 11.4%). Further, the “GG” genotype of *IL1R1* rs3917225 exhibited increased risk of OA after adjustment by age and gender (Table 3; OR= 2.03, 95%CI: 1.20-3.43, *P*= 0.008).

**Table 2:** Basic characteristics and allele frequencies of the five SNPs

SNP	Gene	Chromosome	Position	Allele	Minor allele frequency		HWE *P* value	OR (95%CI)	*P*^a^
					Case	Control			
rs10490571	IL1R1	2q12.1	102717337	T/C	0.205	0.168	0.409	1.27 (0.95-1.70)	0.108
rs12712127	IL1R1	2q12.1	102726661	G/A	0.248	0.209	0.000	1.25 (0.95-1.64)	0.105
rs956730	IL1R1	2q12.1	102758116	A/G	0.213	0.268	0.769	0.74 (0.57-0.97)	0.028*
rs3917225	IL1R1	2q12.1	102769302	G/A	0.413	0.337	1.000	1.38 (1.09-1.75)	0.007*
rs3917318	IL1R1	2q12.1	102792760	G/A	0.441	0.483	0.064	0.85 (0.67-1.06)	0.148

HWE: Hardy-Weinberg equilibrium; OR: odds ratio; 95%CI: 95% confidence interval.

^a^*P* values were calculated from Pearson Chi-Square test.

*P ≤ 0.05 indicates statistical significance.

**Table 3:** Significant SNPs correlated with knee OA risk

SNPs	Models	Genotype	Cases	Controls	Without adjustment		With adjustment	
					OR (95% CI)	*P* ^a^	OR (95% CI)	*P* ^b^
rs956730 (G>A)	Genotype	GG	182	158	1.00		1.00	
		AG	105	119	0.77 (0.55-1.07)	0.122	0.86 (0.60-1.23)	0.404
		AA	11	20	0.48 (0.22-1.03)	0.059	0.51 (0.23-1.15)	0.106
	Dominant	GG	182	158	1.00		1.00	
		AA+AG	116	139	0.72 (0.52-1.00)	0.053	0.81 (0.58-1.14)	0.227
	Recessive	GG+AG	287	277	1.00		1.00	
		AA	11	20	0.53 (0.25-1.13)	0.099	0.54 (0.25-1.21)	0.135
	Additive	-	-	-	0.73 (0.56-0.96)	0.026*	0.80 (0.60-1.06)	0.118
rs3917225 (A>G)	Genotype	AA	106	131	1.00		1.00	
		GA	138	132	1.29 (0.91-1.83)	0.151	1.32 (0.91-1.91)	0.139
		GG	54	34	1.96 (1.19-3.24)	0.008*	2.03 (1.20-3.43)	0.008*
	Dominant	AA	106	131	1.00		1.00	
		GG+GA	192	166	1.43 (1.03-1.99)	0.033*	1.47 (1.04-2.07)	0.030*
	Recessive	AA+GA	244	263	1.00		1.00	
		GG	54	34	1.71 (1.08-2.72)	0.023*	1.75 (1.08-2.85)	0.023*
	Additive	-	-	-	1.37 (1.09-1.74)	0.008*	1.40 (1.09-1.79)	0.007*

SNP: Single nucleotide polymorphism; OR: odds ratio; 95%CI: 95% confidence interval.

^a^*P* values were calculated from unconditional logistic regression analysis.

^b^*P* values were calculated by unconditional logistic regression analysis with adjustments for gender and age.

*P ≤ 0.05 indicates statistical significance.

### Association between IL1R1 polymorphisms and knee OA risk using multiple genetic models and haplotype analysis

We evaluated the correlation between the *IL1R1* SNPs and knee OA susceptibility using multiple genetic models by hypothesizing that the minor allele of each polymorphism was a risk factor (Table 3). We observed that irrespective of age and gender, *IL1R1* rs3917225 was linked to an increased risk of knee OA based on the results of the dominant model (adjusted OR= 1.47, 95%CI: 1.04-2.07, *P*= 0.030), the recessive model (adjusted OR= 1.75, 95%CI: 1.08-2.85, *P*= 0.023), and the additive model (adjusted OR= 1.40, 95%CI: 1.09-1.79, *P*= 0.007). On the other hand, *IL1R1* rs956730 was correlated with a decreased risk of OA under the additive model (OR= 0.73, 95%CI: 0.56-0.96, *P*= 0.026), but was insignificant when the data was adjusted by age and gender in multivariate unconditional logistic regression analysis. Further, we constructed haplotypes of *IL1R1* and analyzed the risk of knee OA in regard to these. However, we failed to identify any pairwise linkage disequilibrium for the five SNPs in *IL1R1*.

## DISCUSSION

In this study, we genotyped five polymorphisms in the *IL1R1* gene and assessed their correlations with knee OA susceptibility in the Northwestern Chinese Han population. Our data showed that *IL1R1* rs3917225 increased OA risk whereas *IL1R1* rs956730 decreased predisposition to OA.

Various studies have shown the effect of the *IL-1* gene cluster on severe hand, knee, and Hip OA [12, 13, 15, 16].e Dinarello et al. found that IL1R1, the high affinity receptor of IL-1 was not abundantly expressed on the cell surface [17]. Our data was in accordance with previous findings that the affected In this study, we postulated that the changes of affinity and the number of IL1R1 resulting from the SNPs (rs956730 and rs3917225) may alter the combination of IL1R1 and IL-1 on the cell surface significantly and hence modulate the inflammatory processes associated with joint destruction accordingly. This assumption was in accordance with previous findings that the degree of *IL1R1* expression on the cell surface affected the response of cells to IL-1 [18].

Our study is the first to associate genetic polymorphisms of *IL1R1* with knee OA risk, based on the Northwestern Chinese Han population. Nakki and others showed evidence for the relevance of *IL1R1* polymorphisms in a Finnish population for severe hand OA by using single SNP association analysis [14]. However, Solovieva and others did not find any correlation of *IL1R1* polymorphisms with bilateral distal interphalangeal joint OA in a Finnish population using single polymorphic markers [19]. Also, Smith and colleagues did not find any association between individual *IL1R1* polymorphisms and haplotypes with knee OA risk in a British study [13]. Differences in these studies could be either due to ethnic differences among the subjects analyzed or due to varying effects of the *IL1R1* polymorphisms in the pathogenesis of knee and hand joint OA or varying sample sizes [20]. Therefore, several limitations must be considered to interpret the results of our study. First, the sample size of our study was relatively small (298 cases and 297 controls). Second, although variations in age and gender were considered during our analysis, we could not statistically analyze other variables like body mass and occupation due to lack of these data from both patients and controls.

In summary, our study highlights the association of genetic polymorphisms of *IL1R1* with knee OA susceptibility in the Northwestern Chinese Han population.

## MATERIALS AND METHODS

### Study subjects

This study was conducted in accordance with the criteria listed in the Helsinki Declaration and was approved by the ethics committee of the Second Affiliated Hospital of Inner Mongolia Medical University and Honghui Hospital. 298 knee OA cases and 297 controls were enrolled in the Second Affiliated Hospital of Inner Mongolia Medical University and Honghui Hospital from January 2013 to January 2016. Inclusion and exclusion criteria of cases were as follows: (1) Subjects recruited in this study were all from the ethnically homogeneous Northwestern Chinese Han population. (2) The diagnosis of knee OA conformed to the criteria of the American College of Rheumatology [21]. (3) All subjects that suffered from severe trauma of knee joint or rheumatoid arthritis were excluded. The controls were selected in parallel with the cases from the physical examination center with criteria of not having a personal or a family history of knee OA. Informed consent was obtained from all participants after fully explaining the details of our study.

### SNP genotyping

Using the Hapmap database, five candidate polymorphisms (rs10490571, rs12712127, rs956730, rs3917225, and rs3917318) in *IL1R1* (minor allele frequencies > 5%) were selected for genotyping in the case study subjects and controls from the Chinese Han population. Of these, two variants (rs956730 and rs3917225) have previously been investigated for hand OA. The other three SNPs were chosen randomly and have not been reported for OA susceptibility. Peripheral blood from all participants was collected into EDTA containing tubes.ic Genomic DNA was isolated from leukocytes of the blood samples using the GoldMag-Mini Purification Kit (GoldMag Co. Ltd. Xi’an city, China). Genomic DNA was quantitated using NanoDrop 2000 (Thermo Scientific, Waltham, Massachusetts, USA) at a wavelength value of A260 and A280 nm. Multiplexed SNP MassEXTEND assay was designed using the Sequenom MassARRAY Assay Design 3.0 Software. SNP genotyping was performed with Sequenom MassARRAY RS1000 (Sequenom, SanDiego, CA). The primers used to identify the *IL1R1* polymorphisms are listed in Table 4. Finally, we processed all data using Sequenom Typer 4.0 Software (Sequenom Co. Ltd) [22].

**Table 4:** Primers used for identification of the IL1R1 polymorphisms

SNP	First PCRP(5′→3′)	Second PCRP(5′→3′)	UEP SEQ(5′→3′)
rs10490571	ACGTTGGATGTAGAAAGCTGGACACAGTGC	ACGTTGGATGCCTGGCTGCTTATCATACTC	AGGCAATGATACATGAACAATTC
rs12712127	ACGTTGGATGCTTCCACCTCTTTTGCACTC	ACGTTGGATGAAGAGGCAGAAAATGCACCG	gagACAGCTATGGATCAAGGTA
rs956730	ACGTTGGATGGGCTCAGGTTACCTCAATTC	ACGTTGGATGAGGCTCTTGTTCTCGTAACC	CCCTGGATATGCCTCTT
rs3917225	ACGTTGGATGAACACACCTCTGATACCTTG	ACGTTGGATGCAGCCTGACTAGTTCAACAC	gacCTAAATCCCAAGCTATTATTCAC
rs3917318	ACGTTGGATGGCCATACGGTTGTGAAAAGC	ACGTTGGATGGTCTGAAAAACAGGAAGCAC	GTAAGTAAAATTCTATTTATCATCATTC

SNP, single nucleotide polymorphism; PCRP, PCR primer; UEP, Unextended mini sequencing primer.

### Statistical analysis

Statistical analysis was conducted using Microsoft Excel and SPSS 16.0 (SPSS, Chicago IL USA). Pearson Chi-Square test and Welch's *t* test were used to detect differences in gender and age between patients and controls, respectively. Allele frequencies of SNPs in controls were evaluated by the exact test to determine whether these polymorphisms departed from Hardy-Weinberg equilibrium (HWE). The Pearson Chi-Square test was used to compare the differences in SNPs genotype and allele frequencies between patients and controls. The relationship between each variant and knee OA risk was determined under dominant, recessive, and additive genetic models. Multivariate unconditional logistic regression analysis was performed to test for significance after adjustment for gender and age. Haplotype construction and linkage disequilibrium was analyzed and visualized using the SHEsis software platform [23]. Odds risk (OR) and 95% confidence interval (CI) values were calculated by unconditional logistic regression models with or without adjustment for gender and age and used to assess the relationship between each of the SNPs and knee OA susceptibility. A two-sided *P*≤ 0.05 was considered as statistically significant.
